# Plasma Clot Lysis Time and Its Association with Cardiovascular Risk Factors in Black Africans

**DOI:** 10.1371/journal.pone.0048881

**Published:** 2012-11-08

**Authors:** Zelda de Lange, Marlien Pieters, Johann C. Jerling, Annamarie Kruger, Dingeman C. Rijken

**Affiliations:** 1 Centre of Excellence for Nutrition, North-West University, Potchefstroom, South Africa; 2 Africa Unit for Transdisciplinary Health Research, North-West University, Potchefstroom, South Africa; 3 Department of Haematology, Erasmus University Medical Centre, Rotterdam, The Netherlands; Maastricht University Medical Center, The Netherlands

## Abstract

Studies in populations of European descent show longer plasma clot lysis times (CLT) in patients with cardiovascular disease (CVD) than in controls. No data are available on the association between CVD risk factors and fibrinolytic potential in black Africans, a group undergoing rapid urbanisation with increased CVD prevalence. We investigated associations between known CVD risk factors and CLT in black Africans and whether CLTs differ between rural and urban participants in light of differences in CVD risk.

Data from 1000 rural and 1000 urban apparently healthy black South Africans (35–60 years) were cross-sectionally analysed.

Increased PAI-1_act_, BMI, HbA1c, triglycerides, the metabolic syndrome, fibrinogen concentration, CRP, female sex and positive HIV status were associated with increased CLTs, while habitual alcohol consumption associated with decreased CLT. No differences in CLT were found between age and smoking categories, contraceptive use or hyper- and normotensive participants. Urban women had longer CLT than rural women while no differences were observed for men.

CLT was associated with many known CVD risk factors in black Africans. Differences were however observed, compared to data from populations of European descent available in the literature, suggesting possible ethnic differences. The effect of urbanisation on CLT is influenced by traditional CVD risk factors and their prevalence in urban and rural communities.

## Introduction

Cardiovascular disease (CVD) is a global problem and CVD risk factors and disease rates continue to rise [1]. The development of CVD may start early in life but the immediate underlying cause of a CVD event is the occlusion of a critically situated blood vessel by a blood clot which results in loss of blood flow to vital organs [2]. Therefore, the optimal breakdown of clots is an important protective mechanism in CVD [3].

Various proteins are involved in the lysis of blood clots. These proteins, such as plasminogen activator inhibitor type-1 (PAI-1), tissue-type plasminogen activator (tPA) and plasminogen can be measured individually. They then serve as proxy markers for fibrinolysis. Alternatively one can measure the global ability of blood or plasma to lyse clots, with the use of global fibrinolytic assays. These assays give an indication of the speed with which the body can lyse clots, often reported as lysis time. In recent years a plasma fibrinolytic potential assay that mimics the physiological initiation of coagulation by tissue factor and clot breakdown by tPA from the endothelium was developed [4].

Various studies conducted in populations of European descent have shown plasma clot lysis times (CLT) to be associated with CVD. In general, CVD patients had longer CLTs than controls [5]–[9], although it was not the case in all studies [10]. CLT has furthermore been shown to be associated with individual CVD risk factors such as increased body mass index (BMI), diabetes, increased total cholesterol, triglycerides and CRP in European descendant populations [7], [8].

No information is available regarding the association of clot lysis times and CVD risk factors in Africans, an under-studied population in CVD epidemiology. CVD, which has often been thought of as a problem of developed countries is now a major problem of developing countries including South Africa [11], [13]. This increase in CVD prevalence is considered to be attributed to urbanisation of the black South African population due to decreased physical activity and changes to Westernised lifestyle and diet. It has furthermore previously been shown that CVD risk factors and their contribution to CVD risk may differ between blacks and European descendant populations [11], [12]. Two large epidemiological studies in black South Africans have shown various CVD risk factors to increase with urbanisation. Vorster [14] reviewed data from the Transition and Health during Urbanisation in South Africa (THUSA) study and reported BMI, smoking prevalence in men, total serum cholesterol levels and blood pressure to increase with urbanisation. In the Prospective Urban and Rural Epidemiological (PURE) population Pieters et al. [15] found blood pressure, BMI, waist circumference, triglyceride concentrations, fasting plasma glucose, and PAI-1_act_, all factors associated with CVD, to be significantly higher in the urban participants than in the rural group.

The purpose of this study was therefore to determine whether urbanisation, with its increased prevalence of CVD risk factors, is associated with hypofibrinolysis, and secondly to determine the association between CLT and traditional CVD risk factors in black South Africans.

## Materials and Methods

### Study Population

Participants were recruited to take part in the South African arm of the international PURE study. This is a large-scale cohort study that tracks changing lifestyles, risk factors and chronic disease using periodic standardised data collection in rural and urban areas of 17 countries in transition over 12 years [16], [17]. The data reported here are from the baseline data of just over 2000 randomly selected participants from well-established rural (living under tribal law) and urban (living in informal and formal settlements surrounding cities) communities in the North West Province of South Africa. The sample size was decided upon, based on power calculations performed on the THUSA results, which is a cross-sectional epidemiological study, performed on similar communities in the same province 10 years prior to PURE [14]. In order to obtain 2000 participants it was decided to randomly include 6000 households (3000 from rural and urban respectively) based on previous experience from the THUSA study. From these 6000 households, 4000 subjects were identified who fitted the inclusion criteria. Of these 4000, 2792 (rural = 1444, urban = 1348) agreed to take part in the study, indicated their availability during the blood collection period and had no plans to relocate in the foreseeable future. During the 12 week blood collection period in 2005 blood was finally collected from 1006 rural and 1004 urban participants. Apparently healthy black South African men and women between the ages of 35 and 60 years were eligible to participate. Use of chronic medication for non-communicable diseases and/or any self-reported acute illness were bases for exclusion. The Ethics committee of the North-West University, South Africa approved this study. The study procedure was explained to participants in their home language, after which participants signed informed consent forms and the study commenced. All data were treated confidentially and all analyses were performed with coded data.

### Blood Collection

Qualified nursing sisters collected fasting blood samples with minimum stasis from the antecubital veins of participants using sterile winged infusion sets and syringes between 07∶00 and 11∶00 on days of data collection. For the analysis of lipids and C-reactive protein (CRP) in serum, blood was collected in tubes without anticoagulant. Blood was collected in EDTA tubes for the determination of glycosylated haemoglobin (HbA1c) and in fluoride tubes for glucose measurements. For the analysis of PAI-1_act_, fibrinogen concentration and plasma fibrinolytic potential, blood was collected into citrate tubes and kept on ice until centrifugation. Samples were centrifuged at 2000×g for 15 minutes at 10°C within 30 minutes of collection. Aliquots were frozen on dry ice, stored in the field at −18°C and then after 2–4 days at −82°C until analysis.

### Laboratory Analysis

Serum lipids and high-sensitivity CRP were measured using a Sequential Multiple Analyser Computer (SMAC), using the Konelab™ autoanalyser (Thermo Fischer Scientific, Vantaa, Finland). HbA1c was determined with the D-10 Haemoglobin testing system (Biorad, Hercules, CA, USA). A hexokinase method using the Synchron®Sytem(s) (Beckman Coulter Co., Fullerton, CA, USA) and reagents was used to measure plasma glucose. Human Immunodeficiency Virus (HIV) status of participants was determined according to the South African Department of Health protocol and UNAIDS/WHO Policy statement on HIV-testing with the Rapid HIV test, and if positive a Pareeshak test was performed to confirm the results. Participants received pre-test counselling and for those who chose to know the results of their HIV test, post-test counselling was done in privacy. PAI-1_act_ was measured using an indirect enzymatic method (Spectrolyze PAI-1, Trinity Biotech, Bray, Ireland). A modified Clauss method (Multifibrin U-test, Dade Behring, Deerfield, IL, USA) on the Dade Behring BCS coagulation analyser was used to determine fibrinogen concentrations. Plasma fibrinolytic potential of tissue factor induced clots, lysed by exogenous tPA was measured over a period of 4 weeks with the method of Lisman et al. [5] with slightly modified tissue factor and tPA concentrations in order to obtain comparable CLTs of about 60 min (intra-assay CV = 3.6%, between plate CV = 4.5%). Final concentrations were tissue factor (125×diluted; Dade Innovin, Siemens Healthcare Diagnostics Inc., Marburg, Germany), CaCl_2_ (17 mmol/l), tPA (100 ng/ml; Actilyse, Boehringer Ingelheim, Ingelheim, Germany) and phospholipid vesicles (10 µmol/l; Rossix, Mölndal, Sweden). CLT was defined as the time from the midpoint of clear to maximum turbidity, which is representative of clot formation, to the midpoint of maximum turbidity to clear, which represents the lysis of the clot [5].

### Dietary Intake Analysis and Anthropometrical Measurements

Quantitative Food Frequency questionnaires, designed and validated for use in this population, were used to determine habitual alcohol consumption of study participants.

Anthropometrical measurements were taken according to the International Standards of Anthropometric assessment [International society for the advancement of Kinanthropometry] and included weight and height as well as waist circumference. The recommended waist circumference cut-off for central obesity in Sub-Saharan Africans, which is ≥94 cm for men and ≥80 cm for women [18] were used to define central obesity and used as the cut-off for Metabolic syndrome criteria. Blood pressure was measured with subjects sitting relaxed but upright and the right arm supported at heart level using an automatic digital blood pressure monitor (Omron HEM-757).

### Statistical Analysis

Data were analysed with the computer software package Statistica (Statsoft Inc., Tulsa, Oklahoma, USA). A p-value of 0.05 or less was regarded as statistically significant. Normally distributed data are reported as mean (95% confidence interval or SD). Data that were not normally distributed were log transformed to improve normality and reported as median (25^th^ –75^th^ percentile). T-tests for independent samples were used when comparing parametric data between two groups and analysis of variance (ANOVA) with Tukey’s Honest Significant Difference post hoc test were used for comparisons between three or more groups. The Mann-Whitney U test was used for comparison of non-parametric data between 2 groups. Analysis of co-variance (ANCOVA) was used when comparisons between groups required adjustment. Mean differences with corresponding 95% confidence intervals are also reported. CLT was not significantly different between women who used contraceptives and those who did not, so we did not stratify or adjust for contraceptive use. Forward Stepwise Multiple Regression analysis was used to determine the main contributors to the variance in CLT in the PURE population using parametric and log transformed data.

## Results

### General Characteristics of Rural and Urban Participants

Table 1 provides population characteristics for the total study population as well as for the urban and rural groups separately. Sex differences are also indicated for variables with sex specific cut-offs. CLT could be determined for 1802 participants only due to inadequate sample volume and/or haemolysis of some samples. Baseline characteristics of the 1802 participants did not differ from that of the total group. The mean CLT was 57.3 (±11.2) minutes (Table 1). CLT was longer in urban than in rural women (mean difference 1.79 min, 95% CI 0.60–2.99), while no difference was observed for men before adjustments. Taking the standard deviation into consideration, the likelihood of this difference being clinically significant is small. Urban participants had significantly higher blood pressure, BMI (women only), waist circumference (women only), triglycerides, PAI-1_act_ and fasting blood glucose than the rural participants. While urban women consumed more alcohol than rural women, the opposite was seen for men. Fibrinogen concentration and CRP were, however higher in the rural participants.

**Table 1:** Characteristics of the South African PURE study population.

Variable	Total population (n = 2010)	Urban (n = 1004)	Rural (n = 1006)	Rural vs. urban p-value
Age (years)	48 (41–56)	48 (42–57)	47 (41–55)	0.0002
Men/women n (%)	749 (37.3)/1260 (62.7)	401 (39.9)/602 (60.0)	348 (34.6)/658 (65.4)	0.01
CLT (minutes)	57.3±11.2	57.6±12.0	57.0±10.5	0.09
Men	52.9±11.6*	52.6±12.4*	53.3±10.6*	0.55
Women	59.9±10.2*	60.8±10.5*	59.0±10.0*	0.015
SBP (mm/Hg)	133.5±24.5	137.3±25.1	129.7±23.3	<0.0001
DBP (mm/Hg)	87.7±14.5	89.3±14.5	86.2±14.5	<0.0001
BMI (kg/m^2^)	22.9 (19.3–28.6)	23.4 (19.5–29.4)	22.4 (19.1–28.1)	<0.0001
Men	19.8 (18.1–22.4)	20.0 (18.3–22.8)	19.7 (18.0–22.2)	0.60
Women	25.8 (21.4–31.7)	27.1 (22.3–32.5)	24.9 (20.8–30.7)	<0.0001
Waist circumference (cm)	77.5 (70.2–87.7)	78.5 (70.9–89.0)	76.0 (69.7–86.9)	0.001
Men	74.4 (69.9–81.3)*	74.3 (69.7–81.8)*	74.5 (70.2–80.5)*	0.49
Women	81.1 (70.6–91.3)*	82.8 (73.1–92.8)*	78.8 (69.5–89.5)*	<0.0001
HDL-cholesterol (mmol/l)	1.52±0.63	1.52±0.65	1.52±0.62	0.83
Men	1.58±0.66*	1.61±0.66*	1.55±0.66	0.22
Women	1.48±0.62*	1.46±0.63*	1.50±0.61	0.17
Triglycerides (mmol/l)	1.08 (0.82–1.55)	1.11 (0.84–1.65)	1.05 (0.80–1.43)	<0.001
Total cholesterol (mmol/l)	4.82 (4.01–5.87)	4.89 (4.00–5.97)	4.75 (4.02–5.80)	0.32
LDL-cholesterol (mmol/l)	2.79 (2.08–3.65)	2.81 (2.07–3.66)	2.77 (2.09–3.63)	0.95
PAI-1 (U/ml)	4.26 (1.27–7.92)	5.01 (1.76–9.11)	3.58 (0.81 6.85)	<0.0001
Fibrinogen (g/l)	2.90 (2.30–5.00)	2.70 (2.20–4.30)	3.00 (2.40–5.40)	<0.0001
Men	2.60 (2.10–3.70)*	2.50 (2.00–3.30)*	2.80 (2.20–4.30)*	0.04
Women	3.10 (2.30–5.50)*	2.90 (2.30–5.40)*	3.20 (2.50–5.70)*	<0.001
HbA1c	5.50 (5.30–5.80)	5.50 (5.20–5.80)	5.60 (5.30–5.80)	0.89
Fasting plasma glucose (mM)	4.80 (4.30–5.30)	4.90 (4.30–5.40)	4.70 (4.40–5.20)	0.06
CRP (mg/l)	3.29 (0.96–9.34)	3.25 (1.12–9.85)	3.33 (0.85–9.02)	0.07
Alcohol consumption (g/day)				
(Alcohol consumers only)				
Total group (n = 872)	15.4 (6.43–34.7)	15.9 (7.71–30.9)	15.0 (5.14–44.8)	0.96
Men (U n = 281, R n = 186)	19.7 (7.71–40.0)*	19.3 (11.4–34.7)*	21.9 (5.86–56.6)*	0.88
Women (U n = 252, R n = 153)	13.4 (4.29–30.9)*	14.8 (4.86–26.8)*	11.4 (3.21–38.6)*	0.92

Parametric data reported as mean ± SD and non-parametric data as median (25^th^-75^th^ percentile); SBP, systolic blood pressure; DBP, diastolic blood pressure; BMI, body mass index, HDL, high density lipoprotein; LDL, low-density lipoprotein, PAI-1, plasminogen activator inhibitor type-1; HbA1c, glycosylated haemoglobin; CLT, clot-lysis time; R rural; U, urban; *Means differed significantly between sexes; rural vs. urban p-value adjusted for age and sex, and only for age where values are reported for men and women separately.

### Associations between CLT and CVD Risk Factors

The associations between CLT and various non-biochemical cardiovascular risk factors are presented in Table 2. ANCOVA’s were used to adjust for factors that could potentially influence/obscure independent associations or for intermediate variables that could, at least in part, explain the associations. These include variables that themselves were associated with CLT and which differed between the respective sub-categories. There was no significant difference in CLT between different age categories, contraceptive use or between hyper- and normotensive participants. Women had significantly longer CLTs than men (mean difference 1.3 min 95%CI 0.27–2.38), also after adjustment for differences in BMI and PAI-1_act_ (which differed between men and women, and which were associated with CLT)_._ CLTs were significantly increased with increasing BMI categories (mean difference between lowest and highest BMI categories: 13.8 min, 95%CI 12.2–15.4) and in participants with abdominal obesity (mean difference 9.3 min, 95%CI 8.31–10.3). These differences were also likely to be clinically significant, taking the standard deviations into consideration. Although CLT correlated with both waist circumference (r = 0.42, p<0.0001) and BMI (r = 0.47, p<0.0001), the correlation with BMI was stronger and BMI was used in further analyses. Since sex differences were present amongst the BMI and waist circumference categories, we adjusted for sex, but significance remained. Participants who were diagnosed with the metabolic syndrome, using the criteria recommended by Alberti et al. [18] also had significantly longer CLTs than those without metabolic syndrome (mean difference 4.1 min 95% CI 2.84–5.36). Current smokers had significantly shorter CLTs than non-smokers (mean difference 4.5 min 95% CI 3.48–5.52). This significance remained after adjustment for BMI, but disappeared after adjustment for habitual alcohol consumption (which differed between smoking categories). Participants who were non-drinkers had significantly longer CLTs than participants who reported to be moderate (mean difference 5.2 min 95% CI 4.03–6.37) or heavy drinkers (mean difference 6.9 min 95% CI 5.41–8.39). Non-drinkers had significantly higher waist circumference measures (also associated with longer CLT) and significantly lower PAI-1_act_ (associated with shorter CLTs) than drinkers, therefore we adjusted for both, but significance remained (p<0.0001). Men drank significantly more than women, and since sex differences for CLT were found, we also adjusted for sex, but again significance remained (p<0.0001).

**Table 2:** Non-biochemical cardiovascular disease risk factors and their association with CLT.

Variable	N	Mean CLT (95% CI)minutes	ANCOVA p-value: Model 1^•^ (Model 2)^Δ^ variables adjusted for	Mean difference (95% CI)
**Age groups**				
30–39 years	332	56.9 (55.9–57.9)	0.71	0.8 (−0.95–2.55)
40–49 years	700	57.5 (56.7–58.3)	(0.56) Smoking, alcohol	
50–59 years	482	57.0 (55.9–58.0)	consumption and BMI	
≥60 years	288	57.7 (56.3–59.2)		
**Sex**			<0.0001	
Men	670	52.9 (52.1–53.9)	(<0.0001) BMI	1.3 (0.27–2.38)
Women	1132	59.9 (59.3–60.5)	(0.003) PAI-1act, BMI	
**Contraceptive use**				
Yes	403	60.5 (59.5–61.6)	0.10	1.1 (−0.18–2.38)
No	667	59.4 (58.7–60.2)		
**Blood pressure**				
Normal	946	57.3 (56.6–58.0)	0.93	0 (−1.05–1.05)
Hypertensive	842	57.3 (56.5–58.1)		
**BMI**				
<18.5 kg/m^2^	314	50.8 (49.6–52.0)^†^	<0.0001	13.8 (12.2–15.4)
18.5–24.9 kg/m^2^	739	55.3 (54.5–56.0)^†^	(0.0001) PAI-1act	
25–29.9 kg/m^2^	297	60.8 (59.6–61.9)^†^	(<0.001) PAI-1act, sex	
≥30 kg/m^2^	354	64.6 (63.6–65.6)^†^		
**Waist circumference**			<0.0001	
Normal	1168	54.1 (53.5–54.7)^†^	(<0.0001) PAI-1act	9.3 (8.31–10.3)
Abdominal obesity	617	63.4 (62.6–64.2)^†^	(<0.0001) PAI-1act, sex	
**Metabolic syndrome**				
Yes	434	60.4 (59.2–61.5)^†^	<0.0001	4.1 (2.84–5.36)
No	1358	56.3 (55.7–56.9)^†^		
**Metabolic Syndrome risk score**				
0	278	56.4 (55.1–57.6)	<0.0001	5.5 (3.81–7.19)
1	604	54.9 (54.0–55.8)		
2	476	58.0 (57.1–59.0)		
≥3	434	60.4 (59.2–61.5)		
**Smoking status**				
Yes	943	55.3 (54.6–56.1)^†^	<0.0001	4.5 (3.48–5.52)
No	785	59.8 (59.0–60.5)^†^	(0.016) BMI	
Former	66	56.0 (52.9–59.0)	(0.78) BMI, alcohol	
**Alcohol consumption**			<0.001	
Non-drinkers	964	60.0 (59.4–60.7)^†§^	<0.0001) Sex, WC	6.9 (5.41–8.39)
Moderate drinkers*	467	54.8 (53.9–55.8)^†^	(<0.0001) Sex, WC,	
Heavy drinkers**	320	53.1 (51.7–54.4)^§^	PAI-1act,	

* Moderate drinking: >0<15 g/day for women; >0<30 g/day for men; **Heavy drinking: ≥15 g/day for women; ≥30 g/day for men; ^†‡§‖^Means with the same symbol differed significantly; ^¶^Mean differed significantly from other means in subgroup. WC: waist circumference; BMI: Body mass index; PAI-1: Plasminogen activator inhibitor type-1. For groups with more than two subgroups the difference between the highest and lowest value are reported. N varies among variables due to lack of sample availability that occurred randomly during sample collection. ^•^Model 1: adjusted for age; ^Δ^Model 2: adjusted for age as well as variables indicated in table.

In Table 3 associations between CLT and biochemical CVD risk factors are presented. CLT increased significantly over HbA1c quartiles (mean difference between lowest and highest quartiles: 8.7 min 95% CI 7.38–10.0) and fasting glucose categories (mean difference 4.5 min 95% CI 2.97–6.03). Since a positive association between both HbA1c and fasting plasma glucose with BMI was found, we adjusted for BMI, but the significance between the HbA1c quartiles and fasting glucose quartiles remained. Furthermore significantly longer CLTs were observed in participants with increased serum triglycerides compared to normal triglyceride levels (mean difference 7.6 min 95% CI 6.29–8.91), increased total cholesterol levels compared to normal cholesterol (mean difference 3.5 min 95% CI 2.44–4.56) and decreased HDL-cholesterol levels compared to the recommended values (mean difference 5.8 min 95% CI 4.70–6.90). CLT also increased significantly over the PAI-1_act_ (mean difference between lowest and highest quartiles 15.4 min 95% CI 14.0–16.8), fibrinogen (mean difference between lowest and highest quartiles 4.9 min 95% CI 3.43–6.37) and CRP quartiles (mean difference between lowest and highest quartiles 6.9 min 95% CI 5.42–8.38). Taking the standard deviation into consideration, these differences were all likely to be clinically significant. Significance remained for fibrinogen after adjustment for CRP and significance also remained for CRP after adjustment for PAI-1_act_ and fibrinogen. CLTs tended to be longer in HIV+ compared to HIV- participants (mean difference 1.3 min 95% CI 0.11–2.49). Because there is a difference in BMI between HIV+ and HIV- participants (data not shown) and CLT correlated with BMI we adjusted for BMI. After the adjustment, HIV+ participants now had significantly longer CLTs than the HIV- participants (59.7 vs. 56.9 minutes).

**Table 3:** Biochemical cardiovascular disease risk factors and their association with CLT.

Variable	N	Mean CLT (95% CI)minutes	ANCOVA p-value: Model 1^•^ (Model 2)^Δ^ variables adjusted for	Mean difference(95% CI)
**HbA1c**				
<5.3	443	53.1 (52.2–54.0)^†^	<0.0001	8.7 (7.38–10.0)
≥5.3–<5.5	289	54.1 (52.9–55.3)^†^	(<0.0001) BMI	
≥5.5–<5.8	490	57.8 (56.8–58.8)		
≥5.8	564	61.8 (60.9–62.8)		
**Fasting glucose**				
≤5.5 mmol/l	1435	56.5 (55.9–57.0)	<0.0001	4.5 (2.97–6.03)
>5.5 mmol/l	297	61.0 (59.6–62.5)	(0.003) BMI	
**Triglycerides**				
<1.7 mmol/l	1417	55.8 (55.2–56.3)^†^	<0.0001	7.6 (6.29–8.91)
≥1.7 mmol/l	342	63.4 (62.2–64.6)^†^		
**Total cholesterol**				
<5.2 mmol/l	1059	55.8 (55.2–56.5)^†^	<0.0001	3.5 (2.44–4.56)
≥5.2 mmol/l	710	59.3 (58.5–60.1)^†^		
**HDL-cholesterol**				
Men >1, women>1.2 mmol/l	1233	55.5 (54.9–56.1)^†^	<0.0001	5.8 (4.70–6.90)
Men<1, women<1.2 mmol/l	536	61.3 (60.4–62.2)^†^		
**PAI-1act**				
<1.27 U/ml	456	50.2 (49.3–51.2)^†^	<0.0001	15.4 (14.0–16.8)
≥1.27–<4.26 U/ml	460	54.9 (54.1–55.7)^†^	(<0.0001) Sex	
≥4.26–<7.92 U/ml	446	58.7 (57.9–59.5)^†^		
≥7.92 U/ml	440	65.6 (64.5–66.7)^†^		
**Fibrinogen**				
<2.3 g/L	471	55.1 (54.0–56.1)^†‖^	<0.0001	4.9 (3.43–6.37)
≥2.3–<2.9 g/L	380	56.3 (55.2–57.4)^‡§^	(<0.001) CRP	
≥2.9–<5 g/L	406	58.7 (57.6–59.8)^†§^		
≥5 g/L	421	60.0 (59.0–61.0)^‡‖^		
**CRP**				
<0.964 mg/L	431	53.8 (52.8–54.7)^†^	<0.0001	6.9 (5.42–8.38)
≥0.964–<3.286 mg/L	443	56.2 (55.2–57.1)^†^	(<0.0001) PAI-1act	
≥3.286–<9.340 mg/L	442	58.3 (57.3–59.4)^†^	(<0.0001) Fibrinogen	
≥9.340 mg/L	445	60.7 (59.5–61.8)^†^	(<0.0001) PAI-1act, fibrinogen	
**HIV status**				
Positive	306	58.4 (57.4–59.4)^†^	0.052	1.3 (0.11–2.49)
Negative	1486	57.1 (56.5–57.7)^†^	(<0.0001) BMI	

†‡§‖ Means with the same symbol differed significantly. HDL-cholesterol: high density lipoprotein cholesterol; PAI-1: Plasminogen activator inhibitor type-1; CRP: C-reactive protein; HbA1c: Glycosylated haemoglobin; HIV: Human Immunodeficiency Virus. For groups with more than two subgroups the difference between the highest and lowest value are reported. N varies among variables due to lack of sample availability that occurred randomly during sample collection. ^•^Model 1: adjusted for age; ^Δ^Model 2: adjusted for age as well as variables indicated in table.

In order to determine the main contributors to the variance of CLT the variables in Table 2 and 3 were included in a forward stepwise regression model. The model explained 45% of the variance in CLT. PAI-1_act_ explained 27% of the variance, while BMI, alcohol consumption and HbA1c explained 8%, 3% and 2% respectively. Triglycerides, CRP, HDL-cholesterol and HIV status each explained only 1% of the variance. Blood pressure, total cholesterol, fibrinogen, smoking and age each explained less than 0.5%. In order to prevent inter-correlation between the variables BMI but not waist circumference and HbA1c but not fasting glucose were included. Metabolic syndrome per se was also not included as the individual components were entered into the model separately.

## Discussion

This study investigated for the first time whether urbanisation with its resultant increased CVD risk is associated with hypofibrinolysis. This is also the first paper to investigate the association between known CVD risk factors and global fibrinolytic potential in blacks.

### Rural and Urban Differences for CLT

Clot lysis time was not found to be significantly longer in the urban than the rural participants, despite an increase in most CVD risk factors and positive associations of these risk factors with CLT (which will be discussed below). This can likely be explained through the association of CLT with individual CVD risk factors. CVD risk factors that were increased in the urban group and that are associated with increased clot lysis e.g. triglycerides, PAI-1 and BMI could potentially increase CLT in the urban group. On the other hand some CVD risk factors i.e. fibrinogen and CRP were higher in the rural group and was also found to have positive associations with CLT, therefore contributing to increased CLT in the rural group. Separate analysis for men and women indicated urban women to have a small but statistically significant longer mean CLT than rural women, while no differences were observed for men. A possible reason for rural-urban differences in CLT observed in women and not in men is that the rural-urban differences in the CVD risk factors were more pronounced in the women than the men. This was indeed the case for BMI, for which the rural-urban difference in women was 7 times that of the difference in men. The relative importance of BMI in CLT was additionally established with the multiple regression analysis.

### Association of CLT with CVD Risk Factors

Of the known CVD risk factors investigated, the main contributors to the variance in CLT in this population, as determined with a forward stepwise multiple regression model which included the variables in Tables 2 and 3, were PAI-1_act_ (27%), BMI (8%), alcohol consumption (3%) and HbA1c (2%). A previous study conducted in a population of European descent found triglycerides, BMI, diastolic blood pressure, systolic blood pressure and CRP to be the main contributors to CLT, PAI-1 was, however, not measured [8].

In a separate study investigating the main coagulation and fibrinolytic factors associated with CLT, PAI-1_ag_, in agreement with our PAI-1_act_ results, was found to have the strongest association, explaining 24% of CLT variation in a multiple regression model [19]. The differences in CLT across especially the PAI-1 and BMI quartiles may be of clinical relevance. The difference between the lowest and highest quartiles were around 15 minutes while differences between arterial thrombosis patients and controls in previous studies ranged from 1.9 to 10.8 minutes [7], [8], [19].

Interestingly, in the PURE population CLT correlated better with BMI than with waist circumference even though PAI-1_act_ had a significantly stronger correlation with waist circumference [20]. This stronger correlation of PAI-1 with waist circumference is likely due to the fact that visceral adipose tissue is a major source of PAI-1 and that its levels are further increased by hepatic production in response to adipocyte-derived cytokines [21]–[24]. Adjustment for PAI-1_act_ did not significantly affect the results and CLT remained longer with increased BMI categories and in individuals with abdominal obesity. While PAI-1_act_ seems to be more related to central obesity, CLT seems to be related to general obesity. Although increased PAI-1_act_ levels likely play a major role in this relationship, there seem to be additional mechanisms involved in the association between body fat and CLT, unrelated to the PAI-1 and visceral fat link.

CLT was significantly associated with both glucose and HbA1c. In agreement with these results, Guimarães et al. [7] and Meltzer et al. [6] found white diabetic subjects to have longer CLT than non-diabetic subjects. Another study, however, found no association between CLT and diabetes in control subjects [8]. CLT was also longer in participants diagnosed with the metabolic syndrome, than those without. These results are to be expected since most of the criteria for the metabolic syndrome were all found to be associated with longer CLTs. These results are in agreement with the results of Carter et al. [25], who found CLT to be longer in white patients with the metabolic syndrome, although a different classification system was used to diagnose metabolic syndrome and their clot lysis assay differs from the assay we used.

Although CLT was increased across fibrinogen quartiles, fibrinogen concentration explained less than 0.5% of the variance in CLT and correlated only weakly with CLT (r = 0.18, p<0.0001), indicating that fibrinogen concentration was not one of the main contributing factors of CLT variance in this population. Theoretically fibrinogen could influence fibrin lysis rates through its effect on clot structure as has been demonstrated in purified models [26]–[28]. This effect, however, is less prominent in plasma models likely due to the presence and interaction of other plasma components that also affect clot lysis. It is also possible that other factors included in the model influenced the prediction value of fibrinogen. We also saw a significant increase in CLT across CRP quartiles, also after adjustment for PAI-1 and fibrinogen indicating an independent positive association between inflammation and CLT. These results are in agreement with associations between CLT, fibrinogen and CRP data from the literature [5], [8].

We found no association between CLT and age and between CLT and blood pressure whereas studies in populations of European descent found trends of increased CLT with increased age [5], [7], [8] and increased systolic and diastolic blood pressure [8]. In agreement with this, no association was found between PAI-1_act_ and age in the PURE study population [20].

Our results also show women to have significantly longer CLTs than men, while other studies found, although not significantly, longer CLTs in white men than in women [5], [6], or no differences between men and women [7]. Possible factors that might have explained the longer CLT in women in our study, are PAI-1_act_ and BMI, which were both found to be higher in the women than in the men [20]. Adjustment for PAI-1_act_ and BMI did, however, not significantly affect the results and CLTs remained longer in women than in men, indicating a possible real sex difference in this study population.

We found significantly shorter CLTs for moderate and heavy drinkers than for non-drinkers, while studies in populations of European descent reported no apparent differences between regular users of alcohol and participants who do not use alcohol or do so only occasionally [7], [8]. Three factors that differed significantly between drinkers and non-drinkers [20] and that were found to be associated with CLT were considered in order to explain the association between CLT and alcohol consumption. These factors were sex, waist circumference and PAI-1_act_ [20]. Significant differences in CLT between drinkers and non-drinkers, remained, however after separate adjustment for these three factors. The fact that CLT is shorter in drinkers in this population, while a main factor determining CLT, PAI-1_act_ was found to be increased, suggests that alcohol affects CLT at least in part, in a PAI-1_act_ unrelated manner.

HIV+ participants had longer CLT’s than HIV- participants after adjustments for BMI differences. One would expect the HIV- group, who had the higher BMI values to have longer CLTs due to the link between PAI-1_act_ and adipose tissue or overall body fat. Positive HIV status has on the other hand been shown to be associated with increased PAI-1 antigen and therefore probably impaired fibrinolysis. This increase may be attributed to fat redistribution in patients infected with the HIV virus [29]. However, in the PURE population PAI-1_act_ did not differ between the HIV+ and HIV- participants [30].

Due to the fact that many haemostatic factors play a role and/or influence CLT, the fact that only PAI-1_act_ and fibrinogen concentration were measured in this study population may be a limitation to the interpretation of the results of this study and could potentially lead to residual confounding. Additionally, this being a cross-sectional study, causality could not be determined for CLT. While every attempt has been made to prevent possible selection bias, it is not impossible that it may have occurred in some form.

In conclusion CLT in black Africans associated significantly with many known CVD risk factors. Differences in these associations were however observed, compared to available data from white populations, suggesting possible ethnic differences in the association of CLT with CVD risk. Of the variables measured, CLT was most strongly related to PAI-1_act_ and BMI. CLT seems to be strongly affected by total body fat, but it seems only partly through the PAI-1-visceral fat link. Additional research is required to determine which factors associated with obesity influences CLT. Alcohol consumption in this population was significantly associated with shorter CLT, despite increased PAI-1_act_. This also deserves further attention. Urbanisation per se is not associated with hypofibrinolysis despite an increase in presence of many CVD risk factors. The effect of urbanisation on CLT is dependent on the relationship of the individual CVD risk factors with CLT and to which degree urbanisation affects these risk factors.
